# Editorial

**Published:** 1872-07-01

**Authors:** 


MEDICAL EXAMINER.

*A Semi-Monthly Journal of Medical Sciences.*

EDITED BY

N. S. DAVIS, M. D., AND F. H. DAVIS, M. D.

Chicago, July 1st, 1872.

### EDITORIAL.

DR. H. R. STORER.—This gentleman, who has suffered a severe period of sickness, is now reported recovering.

ETHICAL.—At the recent annual meeting of the Michigan State Medical Society, three female physicians were admitted as members; and a resolution was adopted of the following import: "That the State Medical Society of Michigan adopt as one of its standing rules that members of the Society are strictly prohibited from advertising their business in any way but the following: 'John Doe, M. D., Physician and Surgeon,' or 'Oculist and Aurist,' or any other specialty, at the same time giving the number of office and name of the street on which it is located. If anything further is advertised, the member so offending shall be expelled. Specialists, who advertise as such, are not allowed to engage in general practice."

FOREIGN HONORS.—The degree of Doctor of Civil Law has been conferred by the University of Oxford, England, upon Dr. Samuel D. Gross, of Philadelphia. The Obstetrical Society of London has elected to membership Drs. C. E. Buckingham and W. L. Richardson, of Boston. The King of Sweden has conferred the knighthood of the Royal Order of the Wasa upon Dr. Lewis A. Sayre, of New York.

